# Immediate, but Not Delayed, Microsurgical Skull Reconstruction Exacerbates Brain Damage in Experimental Traumatic Brain Injury Model

**DOI:** 10.1371/journal.pone.0033646

**Published:** 2012-03-16

**Authors:** Loren E. Glover, Naoki Tajiri, Tsz Lau, Yuji Kaneko, Harry van Loveren, Cesario V. Borlongan

**Affiliations:** Department of Neurosurgery and Brain Repair, University of South Florida College of Medicine, Tampa, Florida, United States of America; University of California San Francisco, United States of America

## Abstract

Moderate to severe traumatic brain injury (TBI) often results in malformations to the skull. Aesthetic surgical maneuvers may offer normalized skull structure, but inconsistent surgical closure of the skull area accompanies TBI. We examined whether wound closure by replacement of skull flap and bone wax would allow aesthetic reconstruction of the TBI-induced skull damage without causing any detrimental effects to the cortical tissue. Adult male Sprague-Dawley rats were subjected to TBI using the controlled cortical impact (CCI) injury model. Immediately after the TBI surgery, animals were randomly assigned to skull flap replacement with or without bone wax or no bone reconstruction, then were euthanized at five days post-TBI for pathological analyses. The skull reconstruction provided normalized gross bone architecture, but 2,3,5-triphenyltetrazolium chloride and hematoxylin and eosin staining results revealed larger cortical damage in these animals compared to those that underwent no surgical maneuver at all. Brain swelling accompanied TBI, especially the severe model, that could have relieved the intracranial pressure in those animals with no skull reconstruction. In contrast, the immediate skull reconstruction produced an upregulation of the edema marker aquaporin-4 staining, which likely prevented the therapeutic benefits of brain swelling and resulted in larger cortical infarcts. Interestingly, TBI animals introduced to a delay in skull reconstruction (i.e., 2 days post-TBI) showed significantly reduced edema and infarcts compared to those exposed to immediate skull reconstruction. That immediate, but not delayed, skull reconstruction may exacerbate TBI-induced cortical tissue damage warrants a careful consideration of aesthetic repair of the skull in TBI.

## Introduction

The treatment of traumatic brain injury (TBI) is currently an unmet condition in the United States, afflicting at least 1.7 million people annually, including 275,000 hospitalizations and 52,000 deaths [1]. The economic cost is estimated at $56 billion annually [2]. Due to the economic burden and the lack of therapies, research into the pathophysiology of and the possible treatment for TBI is warranted.

The two most commonly employed animal models of TBI are the fluid percussion model and the controlled cortical impact (CCI) injury model [3]–[6]. The cognitive and motor deficits produced by both models resemble many of the abnormalities observed in the clinical setting [7], [8]. In the present study, the CCI injury model is utilized to create a reproducible TBI in adult, male Sprague-Dawley rats. The initial injury to the brain presents as necrotic cell death in the underlying tissue and as axonal injury in white matter, consistent with clinical pathology. The initial injury is followed by apoptotic cell death in the surrounding tissue due to events such as ischemia, excitotoxicity, and edema [9], [10]. In the CCI injury model, the secondary cell damage extends beyond the cortical tissues such as the adjacent subcortical regions [11], [12].

Penetrating or severe TBI is often accompanied by skull fracture [13]–[17]. Reconstruction of the scalp, skull, and underlying tissues is carried out for aesthetic repair [18], but also to prevent complications (e.g., infections) associated with open head injuries [19] and CSF leakage [20]. In some cases, the skull is reconstructed, i.e., cranioplasty [21], [22]. In other cases, either a craniotomy [23], [24], or craniectomy [25], [26] is performed primarily to reduce intracranial pressure (ICP) [27], [28] and prevent herniation [29], [30]. Decompressive craniectomy allows the brain to swell [31]–[33], in turn lowering ICP [32], [34]. The management of severe TBI undergoing craniectomy remains controversial in the clinical setting, in particular the optimal timing of cranioplasty after decompressive craniectomy is still unknown [35]. Although crainoplasty is typically performed three months after decompressive craniectomy, this skull reconstruction needs to proceed only after brain edema subsides [36]. Moreover, cranioplasty presents with complications such as increased infections [37] and bone flap reabsorption [38], which may hinder the patient's recovery [39]. Civilian and military TBI may present with different skull fractures requiring variable cranioplasty. Whereas 20% of civilian patients diagnosed with TBI underwent cranioplasty after decompressive craniectomy [40], a much higher 45% of TBI soldiers received the same procedures [41]. This large discrepancy in the operative procedure of skull reconstruction after TBI could be due to the severity of battlefield blast explosion-induced TBI that creates cavity injury zones that often extend beyond the border of the exposed surface wound, not commonly observed in civilian TBI [41]. Moreover, the prevalence of complications associated with cranioplasty reveals that although there is no difference in post-operative infection rate (16%–34%), the perioperative infection rate of 12% [41] is much higher in soldiers than civilians (less than 2%; [40], [42]), again likely owing to the disease severity of the military TBI. The current clinical practice emphasizes cranioplasty during the initial hospital admission, in an effort to reduce the overall cost of patient care by eliminating the need for repeat hospital admissions [40]. However, such immediate cranioplasty has not reduced the high overall complication rate of skull reconstruction in TBI [41], [42]. Accordingly, studies are warranted to determine the optimal safety and effective regimen of skull reconstruction in TBI.

Preclinical studies may reveal the potential detrimental effects of skull reconstruction in TBI. Unfortunately, a review of the literature revealed inconsistent skull reconstruction post-TBI in the laboratory setting [21]–[26]. In the present CCI study, immediate skull reconstruction significantly worsened the TBI-induced brain damage accompanied by an upregulation of the edema marker aquaporin-4 compared to TBI animals with no skull reconstruction. A delay in skull reconstruction reduced the edema and prevented the exacerbation of brain damage. Taken together, these results suggest that therapies aimed at reducing cerebral edema should facilitate skull reconstruction in TBI.

## Materials and Methods

### Ethics Statement

The use of animals in this study was conducted in accordance with the recommendations in the Guide for the Care and Use of Laboratory Animals of the National Institutes of Health. The protocol was approved by the Institutional Animal Care and Use Committee (IACUC) at the University of South Florida, College of Medicine. The approved protocol number is 3904R. All efforts were made to minimize animal suffering.

### Surgical Procedures

**Figure 1:**
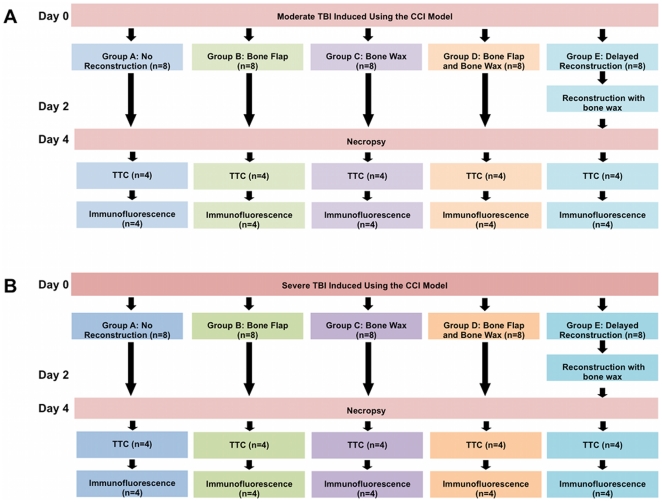
Flowchart of experimental procedures. All animals received moderate (Figure 1A) or severe (Figure 1B) TBI using the CCI model. After the CCI surgery, animals received either no reconstruction (Group A), immediate reconstruction with bone flap (Group B), bone wax (Group C), or bone wax and bone flap (Group D). The animals assigned to Group E in received delayed skull reconstruction on day 2. All animals were euthanized on day 4. Half of the animals (n = 4) from each group were assigned to TTC analysis and the other half (n = 4) were assigned to immunofluorescence assay.

Ten week old, male Sprague-Dawley rats (n = 8 per group) were subjected to TBI using the controlled cortical impact injury model (Pittsburgh Precision Instruments, Inc, USA). Animals were anesthetized using 1–2% isoflurane. Once deep anesthesia was achieved, individual animals were fixed in a stereotaxic frame (David Kopf Instruments, Tujunga, CA, USA), anesthesia was maintained via gas mask. After exposing the skull, a 4.0 mm craniectomy was performed over the left frontoparietal cortex (**−**2.0 mm anteroposterior and +2.0 mm mediolateral to bregma). The pneumatically operated TBI device (with a convex tip diameter of 3.0 mm for rats) impacted the brain at a velocity of 6.0 m/s reaching a depth of 1.0 mm or 2.0 mm for moderate and severe TBI models, respectively, below the dura mater layer and remained in the brain for 150 ms. The impactor rod was angled 15° to the vertical to maintain a perpendicular position in reference to the tangential plane of the brain curvature at the impact surface [3]. A linear variable displacement transducer (Macrosensors, Pennsauken, NJ), which was connected to the impactor, measured velocity and duration to verify consistency. This CCI approach produced skull fracture overlying the frontal cortex. Post-operatively, animals were randomly assigned to skull bone flap replacement with or without bone wax (Lukens), no bone reconstruction or delayed skull reconstruction with bone wax. Delayed skull reconstruction was performed at two days post-TBI in both the moderate and severe TBI models. To reconstruct the skull, the scalp was re-opened and bone wax was applied over the craniectomized region. The bone wax was shaped to conform to the natural contour of the cranium. Animals were euthanized 5 days post-TBI for histological analysis. See Figure 1 for a flowchart of the methods.

### TTC Staining

On day five following surgery, animals were anesthetized and then perfused intracardially with saline. The brain was then removed, immersed in cold saline for 10 min, and sliced in 2.0-mm-thick sections. TBI-induced brain damage was visualized by staining brain slices with 2,3,5-triphenyltetrazolium chloride (TTC), following a procedure previously described [43]. The brain slices were incubated in 2% TTC dissolved in PBS for 20 min at 37°C and then transferred to 4% paraformaldehyde solution for fixation. Brain damage was revealed by a lack of TTC staining, which indicates that tissue is dehydrogenase deficient. The volume of brain damage was measured in each slice and quantified by a computer assisted image analysis system (NIH Image Software, USA) and calculated by the following formula: 2.0 mm (thickness of the slice)×(sum of the damaged area per slice or in all brain slices) then expressed as percentage [43]. To minimize artifacts produced by post-TBI edema in the injured area, the brain damage volume was calculated as described previously [44]. Briefly, the injured area in the ipsilateral hemisphere was indirectly measured by subtracting the non-injured area in the ipsilateral hemisphere from the total intact area of the contralateral hemisphere.

### Immunohistochemistry

The animals utilized for immunohistochemistry were from another cohort of animals. Under deep anesthesia, rats were sacrificed on day 5 after TBI surgery, and perfused through the ascending aorta with 200 ml of cold PBS, followed by 200 ml of 4% PFA in PBS. Brains were removed and post-fixed in the same fixative for 24 hours followed by 30% sucrose in phosphate buffer (PB) for 1 week. Five coronal slices between the anterior edge and posterior edge of the infarct were collected and processed for hematoxylin and eosin (H&E) staining from each brain perfused at day 4. Sections were cut at a thickness of 30 µm by cryostat. The cavity area was defined at the damaged region. Brain sections were observed by using a microscope equipped with a digital camera. Damaged volume in one brain was calculated from the formula: [(area of the damaged region in each section)×0.30] (mm^3^). Infarct volume was then expressed as a percentage of the ipsilateral hemisphere compared to the contralateral hemisphere.

The sections processed for aquaporin-4 immunohistochemistry were from alternating sections of H&E processed brains. Six series of coronal sections were cut at a thickness of 30 µm by cryostat. Free floating sections for immunohistochemistry were incubated overnight at 4°C with an aquaporin-4 monoclonal antibody (1∶100, Abcam) with 5% normal goat serum. After several rinses in PBS, the sections were visualized following the method described above with modification to accelerate FITC with biotin conjugated antimouse IgG antibody and FITC conjugated streptoavidin (1∶500, Sigma, MO). Immunofluorescent microscopy was carried out using a Leica confocal microscope. The density of the aquaporin-4 channels on the ipsilateral cortex was quantified using NIH ImageJ, then compared to the contralateral cortex, and expressed as percentages.

### Statistical Analysis

The data were presented as the mean ± the standard error of the mean (SEM). TTC and H&E infarct volumes and areas, and aquaporin-4 densities were analyzed using single ANOVA followed by post-hoc t-tests. The statistical software used was GraphPad Prism. The level of significance was set at p<0.05.

## Results

### Immediate Skull Reconstruction Provides Normalized Bone Structure in TBI

**Figure 2:**
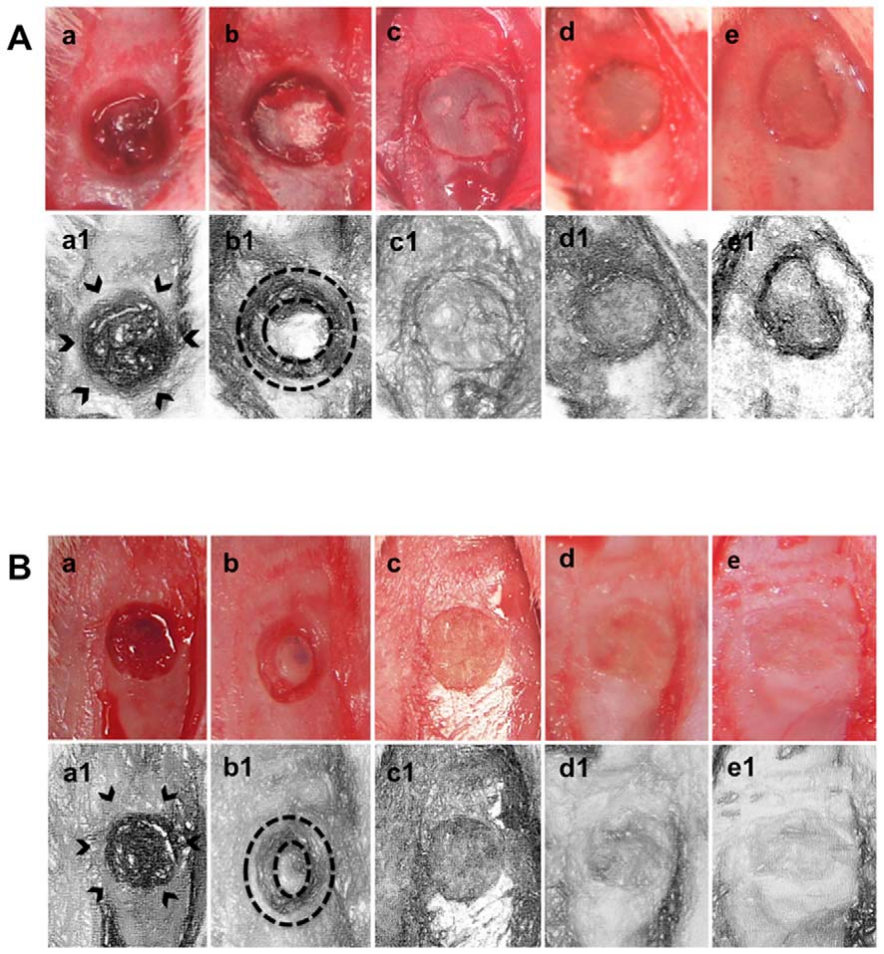
Brain swelling accompanies TBI. Following CCI, obvious brain swelling was detected in animals subjected to no skull reconstruction (Panels a and a1). Reconstruction with bone flap only provided partial skull reconstruction, thereby allowing a tempered brain swelling (Panels b and b1). Skull reconstruction with bone wax only (Panels c and c1), bone wax and bone flap (Panels d and d1), or delayed reconstruction with bone wax (Panels e and e1) afforded normalized skull structure, but also prevented visualization of brain swelling. Arrows are indicative of brain swelling following TBI. Dotted circles show the tempered brain swelling that occurred in the animals that received reconstruction with bone flap only.

Animals exposed to the CCI, especially the severe model, with no reconstruction displayed a skull fracture overlying the frontal cortex (Figure 2). Immediate surgical reconstruction of the skull post-TBI provided normalized bone structure. Reconstruction using only the skull bone flap provided partial reconstruction, whereas reconstruction with bone wax only or bone wax and bone flap afforded almost near complete skull reconstruction. No excessive bleeding, hematoma, or ectopic tissue formation was detected in any of the animals exposed to these skull reconstruction procedures or to no skull reconstruction. Moreover, the immediate aesthetic repair produced physical reconstructions of the skull fracture that were comparable in both moderate and severe TBI models.

### Immediate Skull Reconstruction Exacerbates Cortical Damage Post-TBI

**Figure 3:**
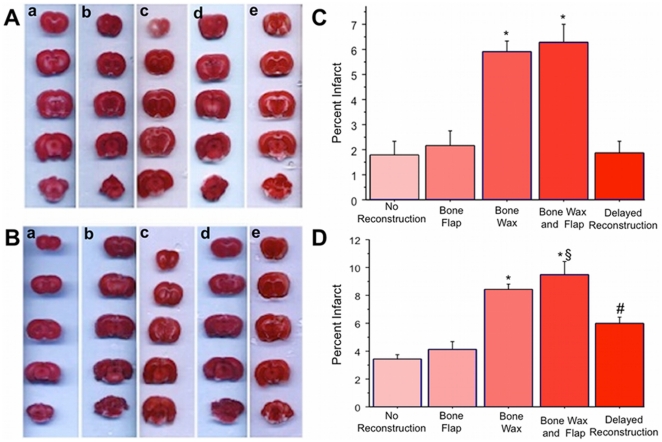
TTC Immediate skull reconstruction with bone wax alone or in combination with bone flap exacerbates cortical damage in TBI. TTC analysis of moderate (Figure 3A, quantified in C) and severe (Figure 3B, quantified in D) TBI revealed that immediate reconstruction with bone wax only or bone wax and bone flap significantly increased cortical damage compared to no reconstruction or reconstruction with only the bone flap. Of interest, delayed reconstruction at 2 days after TBI significantly reduced cortical damage compared to immediate reconstruction with bone wax only or bone wax and bone flap. Bars represent mean ± SEM. Asterisks (*) indicate p<0.05 vs. no reconstruction, immediate reconstruction with bone flap, and delayed reconstruction; # indicates p<0.05 vs. no reconstruction, bone flap, bone wax, and bone wax and flap; § indicates p<0.05 vs. bone wax.

As previously reported [3], [4], the CCI produced cortical damage in adult rats, with the severe model resulting in larger cortical alterations than the moderate model. TTC staining revealed that immediate skull reconstruction, in general, worsened the cortical damage (F_4,15_ = 100.622, p<0.0001 and F_4,15_ = 125.885, p<0.0001 for moderate and severe TBI, respectively) (Figure 3). Although it provided normalized skull structure, TTC staining revealed that immediate reconstruction with bone wax only and combined bone wax and bone flap significantly exacerbated cortical damage compared to the groups that received no skull reconstruction and bone flap only in both the moderate and severe TBI models (p's<0.05) (Figure 3). Although they did not significantly differ in the moderate TBI model, the animals that received bone wax and bone flap displayed significantly larger cortical damage than the bone wax only group in the severe TBI model (p<0.05) (Figure 3). The smaller cortical damage seen in the bone flap treatment after moderate and severe TBI models did not significantly differ from the reduced cortical damage in the no reconstruction group (p's>0.05 for moderate and severe TBI, respectively).

**Figure 4:**
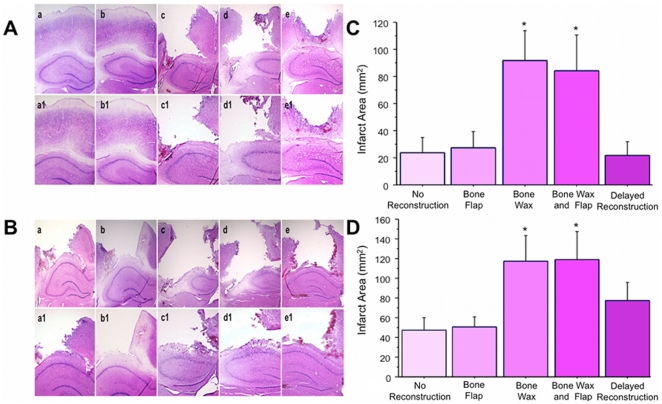
Hematoxylin and eosin staining of moderate (**Figure 4A**, with a–e at 2× magnification, a1–e1 at 4× magnification, and quantified in C) and severe (**Figure 4B**, with a–e at 2× magnification, a1–e1 at 4× magnification, quantified in D) TBI models. The cortical infarcts in the samples that received no reconstruction (Panel a, 2×; Panel a1, 4×) and immediate skull reconstruction with the bone flap only (Panel b, 2×; Panel b1, 4×) were smaller than the infarcts observed in the samples that received skull reconstruction with bone wax only (Panel c, 2×; Panel c1, 4×) and bone wax and bone flap (Panel d, 2×; Panel d1, 4×). The infarcts in the delayed reconstruction group (Panel e, 2×; Panel e1, 4×) were significantly reduced in both moderate and severe TBI models compared to the immediate reconstruction groups that received bone wax or bone wax and bone flap. Bars represent mean ± SEM. Asterisks * indicate p<0.05 vs. no reconstruction, bone flap, and delayed reconstruction.

Hematoxylin and Eosin staining revealed results consistent with the findings from TTC staining. Immediate skull reconstruction worsened cortical damage in both the moderate and severe TBI models (F_4,15_ = 40.213, p<0.0001 and F_4,15_ = 30.109, p<0.0001 for moderate and severe TBI, respectively) (Figure 4). Cortical damage in the animals that received the bone wax only did not significantly differ from the cortical damage in the animals that received both bone wax and bone flap in both the moderate and severe TBI models (p's>0.05) (Figure 4).

### Delayed Skull Reconstruction Does Not Worsen Cortical Damage Post-TBI

Next, we attempted a strategy that might circumvent the worsened brain damage post-TBI produced by immediate skull reconstruction. TTC staining revealed significantly smaller cortical damage in the animals that received delayed reconstruction compared to the animals that received immediate skull reconstruction with bone wax only or combined bone wax and bone flap in both moderate and severe TBI models (p's<0.0001) (Figure 3). In addition, delayed skull reconstruction did not significantly exacerbate cortical damage after the moderate TBI compared to the animals that received no skull reconstruction or reconstruction with bone flap only (p's>0.05) (Figure 3). However, in the severe TBI model, the delayed skull reconstruction group showed significantly larger cortical damage compared to those with no reconstruction or bone flap only (p's<0.0001) (Figure 3).

Hematoxylin and eosin staining revealed that delayed reconstruction significantly reduced cortical damage following moderate and severe TBI compared to animals that received immediate skull reconstruction with bone wax only or combined bone wax and bone flap (p<0.0001 and p<0.01 for moderate and severe TBI models respectively) (Figure 4). Delayed skull reconstruction did not significantly exacerbate cortical damage following moderate and severe TBI compared to the animals that were exposed to no skull reconstruction (p's>0.05) (Figure 4).

### Upregulation of the Edema Marker Aquaporin-4 Accompanies Immediate, but not Delayed Skull Reconstruction

**Figure 5:**
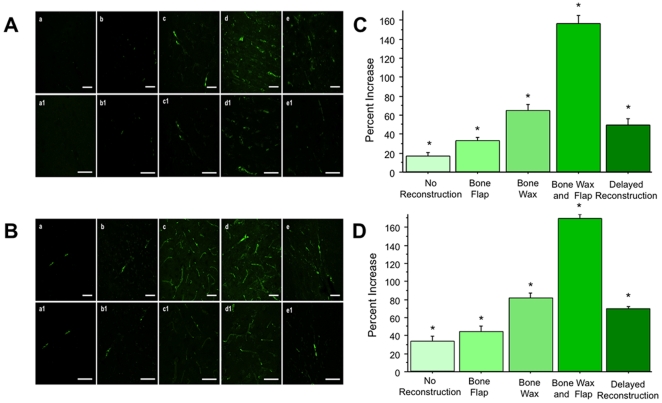
Aquaporin-4 immunofluorescence of moderate (**Figure 5A** **with a–e at 2× magnification, a1–e1 at 4× magnification, and quantified in C) and severe (** **Figure 5B** **with a–e at 2× magnification, a1–e1 at 4× magnification, and quantified in C) TBI brains.** Sparse aquaporin-4 staining was detected in the cortical tissues from TBI animals that received no reconstruction (Panel a, 40×; Panel a1, 60×). A slight, but significant increase in aquaporin-4 staining was seen in immediate skull reconstruction with bone flap (Panel b, 40×; Panel b1, 60×). Widespread aquaporin-4 upregulation was detected in the samples that received immediate skull reconstruction with bone wax only (Panel c, 40×; Panel c1, 60×) and bone wax and bone flap (Panel d, 40×; Panel d1, 60×), which was significantly elevated compared to no skull reconstruction. Aquaporin-4 density was reduced in the samples that received delayed reconstruction compared to immediate reconstruction with bone wax or bone wax and bone flap, but was significantly elevated compared to bone flap only or no reconstruction (Panel e, 40×; Panel e1, 60×). Bars represent mean ± SEM. Asterisks * indicate p<0.05 vs. all other treatment groups. Scale bars represent 50 µm.

Brain swelling was observed almost immediately following CCI, particularly in the severe TBI model. ANOVA revealed treatment effects (F_4,15_ = 394.255, p<0.0001 and F_4,15_ = 520.030, p<0.0001 for moderate and severe TBI, respectively) (Figure 5). Significant upregulation of aquaporin-4 was observed in the TBI animals that received immediate skull reconstruction with bone wax only and bone wax and bone flap in moderate and severe TBI models compared to the animals that received no skull reconstruction or reconstruction with bone flap only (p's<0.0001) (Figure 5). In contrast, TBI animals subjected to delayed skull reconstruction displayed significantly reduced aquaporin-4 levels post-TBI in moderate and severe TBI models compared to immediate skull reconstruction with bone wax and bone flap and bone wax (p's<0.05) (Figure 5). Despite decreased aquaporin-4 expression in the TBI animals that received delayed skull reconstruction, their aquaporin-4 expression remained significantly elevated compared to the TBI animals that received immediate reconstruction with bone flap or no reconstruction in both moderate and severe TBI models (p's<0.01). See Table 1 for comparisons between treatment groups and their corresponding p values.

**Table 1:** Statistics Summary Table.

Moderate TBI			
Comparison	Percent Infarct (TTC)	Infarct Area (H&E)	Aquaporin-4 Quantification
No Reconstruction to Bone Flap	p = 0.8590	p = 0.9941	p = 0.0036*
No Reconstruction to Bone Wax	p<0.0001*	p<0.0001*	p<0.0001*
No Reconstruction to Bone Flap and Bone Wax	p<0.0001*	p<0.0001*	p<0.0001*
No Reconstruction to Delayed Reconstruction	p = 0.9996	p = 0.9997	p<0.0001*
Bone Flap to Bone Wax	p<0.0001*	p<0.0001*	p<0.0001*
Bone Flap to Bone flap and Bone Wax	p<0.0001*	p<0.0001*	p<0.0001*
Bone Flap to Delayed Reconstruction	p = 0.9333	p = 0.9759	p = 0.0024*
Bone Wax to Bone Flap and Bone Wax	p = 0.8740	p = 0.9272	p<0.0001*
Bone Wax to Delayed Reconstruction	p<0.0001*	p<0.0001*	p = 0.0127*
Bone Flap and Bone Wax to Delayed Reconstruction	p<0.0001*	p<0.0001*	p<0.0001*

Severe TBI			
Comparison	Percent Infarct (TTC)	Infarct Area (H&E)	Aquaporin-4 Quantification
No Reconstruction to Bone Flap	p = 0.4546	p = 0.9978	p = 0.0413*
No Reconstruction to Bone Wax	p<0.0001*	p<0.0001*	p<0.0001*
No Reconstruction to Bone Flap and Bone Wax	p<0.0001*	p<0.0001*	p<0.0001*
No Reconstruction to Delayed Reconstruction	p<0.0001*	p = 0.0651	p<0.0001*
Bone Flap to Bone Wax	p<0.0001*	p<0.0001*	p<0.0001*
Bone Flap to Bone flap and Bone Wax	p<0.0001*	p<0.0001*	p<0.0001*
Bone Flap to Delayed Reconstruction	p<0.0001*	p = 0.1158	p<0.0001*
Bone Wax to Bone Flap and Bone Wax	p = 0.0457*	p>0.9999	p<0.0001*
Bone Wax to Delayed Reconstruction	p<0.0001*	p = .0088*	p = 0.0184*
Bone Flap and Bone Wax to Delayed Reconstruction	p<0.0001*	p = .0088*	p<0.0001*

Posthoc pairwise comparisons and their corresponding p-values. Asterisks (*) denotes significance.

## Discussion

The present study demonstrates that immediate skull reconstruction following TBI significantly worsened cortical damage in both the moderate and severe CCI models. This exacerbation of histological deficit was associated with increased edema. In contrast, a delay in skull reconstruction decreased the cortical damage compared to immediate reconstruction, which was accompanied by reduced edema. These observations suggest that an acute window after TBI is critical for facilitation of the therapeutic outcome of brain swelling. Aesthetic skull reconstruction during this early post-TBI period interferes with the natural process of brain remodeling. The delayed skull reconstruction allowed brain swelling to minimize edema, likely by alleviating intracranial pressure. The recognition of this host reparative process should aid in the clinical management of skull fracture in TBI.

Although immediate reconstruction repaired the skull fracture, this early reconstruction with bone wax or bone wax and bone flap significantly increased cortical damage compared to the animals that received no reconstruction in the moderate and severe TBI models. On the other hand, the immediate reconstruction with bone flap did not significantly exacerbate cortical damage compared to the animals that received no reconstruction, likely due to the unfixed bone flap that allowed for partial brain swelling. In an effort to circumvent the increased cortical damage, delayed reconstruction was shown to attenuate the exacerbation of cortical damage compared to immediate reconstruction. Compared to the animals that received no reconstruction, the delayed reconstruction produced comparable (not significant) cortical damage in the moderate, but generated significantly larger cortical damage in the severe TBI model, suggesting that the two-day delay was not sufficient for brain swelling to counteract the intracranial pressure produced by the severe TBI. A prolonged delay post-TBI could allow the maximal therapeutic benefit of brain swelling, via alleviation of intracranial pressure, to abrogate the cortical damage. A limitation of this study is that intracranial pressure following TBI was not directly measured, which will be the topic of subsequent studies.

Reconstruction of the skull following TBI in the laboratory setting is inconsistent. Various methods of skull reconstruction, such as cranioplasty [21], [22], craniotomy [23], [24], and craniectomy [25], [26] have been employed in the laboratory to manage ICP. This study indicates the need for caution in initiating skull reconstruction post-TBI in the laboratory setting, in that immediate reconstruction following TBI may increase cortical damage, which may mask efficacy of potential therapeutics aimed at reducing histological deficits caused by TBI. Various therapies designed to attenutate ICP are utilized in the clinical setting. Decompressive craniectomy is typically employed after therapies like a ventriculostomy or barbiturate-induced coma have failed to lower ICP [32], [34]. The management and timing of skull reconstruction in severe TBI cases remains controversial in the clinic. The present laboratory study demonstrates that a delay in aesthetic reconstruction of the skull is beneficial in reducing histological deficits caused by TBI. In the clinic, cranioplasty may proceed only after alleviating the increased intracranial pressure associated with TBI.

To determine the mechanism underlying the increase in cortical damage after immediate reconstruction, the edema marker aquaporin-4 was evaluated. Immunohistochemical results revealed that aquaporin-4 was significantly increased in TBI animals that received immediate skull reconstruction, but was reduced in those that received delayed skull reconstruction. The TBI animals that received no reconstruction displayed the lowest aquaporin-4 density compared to all treatment groups. The aquaporin-4 density remained elevated in the delayed reconstruction group, suggesting that the two-day delay did not completely abolish edema. A longer interval between TBI and skull reconstruction may further allow reduction in edema. Alternatively, a combination therapy of delayed skull reconstruction and osmotic agents, such as mannitol and hypertonic saline, may allow better control of brain swelling to lower ICP following TBI. A more extensive time course may reveal that longer delay in skull reconstruction will produce similar outcomes as seen in the “no reconstruction” group. However, the current clinical practice indicates cranioplasty when there is the loss of both soft-tissue and skull base support along with the need for cranial vault reconstruction. With this in mind, even if the preclinical data reveal that the “no reconstruction” condition is beneficial in experimental TBI, finding the optimal cranioplasty regimen that is safe and effective will be the logical clinically relevant endpoint.

Brain herniation accompanies the early stages of TBI, and may contribute to the increased tissue damage observed after TBI. Aquaporin-4 is an edema marker that corresponds to a water channel that is specific to astrocytes throughout the central nervous system, particularly the blood-brain barrier (BBB) [45]–[47]. Water entrance through the BBB is considered as a key component to the development of edema post-TBI [48]–[50]. Aquaporin-4 plays a critical role in the regulation of the water content in the brain, thus it may contribute to the exacerbation of tissue damage following experimental TBI [51]. In addition to TBI, cerebral edema has been shown to contribute to the pathophysiology of other central nervous system disorders, including stroke and tumors [52]–[54]. That increased cortical damage occurs via edema exacerbation necessitates the need to find an approach to reduce this adverse side effect of skull reconstruction. Our results suggest that delaying cranioplasty until the TBI-induced cerebral edema has subsided may reduce the unwanted exacerbation of cortical damage associated with this skull reconstruction. The present study is limited to a 4-day post-injury period, but TBI may be accompanied by some delayed pathological manifestations thereby necessitating for subsequent studies to examine long-term effects of skull reconstruction in TBI.

In summary, surgical maneuvers post-TBI provided a normalized skull structure, but also led to increased tissue damage. The upregulation of aquaporin-4 post-TBI indicates increased cerebral edema, which was exacerbated by immediate skull reconstruction. These findings suggest that therapies aimed at regulating aquaporin-4 channels may prove to be a potential therapy for TBI. A delay in skull reconstruction aided in the reduction of aquaporin-4 channels, and in turn ameliorated the TBI-induced cortical damage. The timing of cranioplasty following TBI should be carefully evaluated in both the laboratory and clinical settings.
